# Geriatric or cardiac rehabilitation? Predictors of treatment pathways in advanced age patients after transcatheter aortic valve implantation

**DOI:** 10.1186/s12872-020-01452-x

**Published:** 2020-04-06

**Authors:** Sarah Eichler, Heinz Völler, Rona Reibis, Karl Wegscheider, Christian Butter, Axel Harnath, Annett Salzwedel

**Affiliations:** 1 Department of Rehabilitation Medicine, University of Potsdam, Faculty of Health Sciences Brandenburg, Am Neuen Palais 10, D-14469 Potsdam, Germany; 2 Klinik am See, Rehabilitation Center for Internal Medicine, Rüdersdorf, Germany; 3 Cardiological Outpatient Clinic Am Park Sanssouci, Potsdam, Germany; 4 Department of Medical Biometry and Epidemiology, University Medical Center, Hamburg-Eppendorf, Germany; 5 Heart Center Brandenburg, Medical School Brandenburg, Bernau, Germany; 6 Sana Heart-Center Cottbus, Cottbus, Germany

**Keywords:** TAVI, Treatment pathways, Frailty, Geriatric rehabilitation

## Abstract

**Background:**

Aim of the study was to find predictors of allocating patients after transcatheter aortic valve implantation (TAVI) to geriatric (GR) or cardiac rehabilitation (CR) and describe this new patient group based on a differentiated characterization.

**Methods:**

From 10/2013 to 07/2015, 344 patients with an elective TAVI were consecutively enrolled in this prospective multicentric cohort study. Before intervention, sociodemographic parameters, echocardiographic data, comorbidities, 6-min walk distance (6MWD), quality of life and frailty (score indexing activities of daily living [ADL], cognition, nutrition and mobility) were documented. Out of these, predictors for assignment to CR or GR after TAVI were identified using a multivariable regression model.

**Results:**

After TAVI, 249 patients (80.7 ± 5.1 years, 59.0% female) underwent CR (*n* = 198) or GR (*n* = 51). GR patients were older, less physically active and more often had a level of care, peripheral artery disease as well as a lower left ventricular ejection fraction. The groups also varied in 6MWD. Furthermore, individual components of frailty revealed prognostic impact: higher values in instrumental ADL reduced the probability for referral to GR (OR:0.49, *p* <  0.001), while an impaired mobility was positively associated with referral to GR (OR:3.97, *p* = 0.046). Clinical parameters like stroke (OR:0.19 of GR, *p* = 0.038) and the EuroSCORE (OR:1.04 of GR, *p* = 0.026) were also predictive.

**Conclusion:**

Advanced age patients after TAVI referred to CR or GR differ in several parameters and seem to be different patient groups with specific needs, e.g. regarding activities of daily living and mobility. Thus, our data prove the eligibility of both CR and GR settings.

## Background

Due to the demographic shift and the aging population, the prevalence of aortic stenosis (AS) as the most frequent valve disease is enhancing [1]. For patients having a prohibitive surgical risk, transcatheter aortic valve implantation (TAVI) has been developed as an alternative to the surgical valve replacement. Several clinical trials and registries have demonstrated the advantages and the procedural success of mid- to long-term outcomes and the procedure is now used as a golden standard [2, 3]. Procedural and in-hospital mortality rates could be reduced and therefore, the frequency of catheter-based valve procedures is steadily increasing. It has overtaken the slightly decreased number of surgical procedures in Germany [4].

Consequently, multimorbid octogenarians with functional limitations become more present in cardiac rehabilitation (CR), whereby hospitals have to define the decision making process for the subsequent treatment and rehabilitation centers have to focus the therapy offer on the patients’ individual demands such as improving postural control and combating malnutrition. After TAVI, cardiac rehabilitation already leads to significant improvements in exercise tolerance, walking capacity, muscle strength and quality of life [5–9]. Thus, official position statements promote the implementation of cardiac rehabilitation after TAVI, although there are no specific therapies yet for the individual needs of this old patient group [10, 11].

In Germany, multicomponent CR represents a well-established treatment for the improvement of functional and psychocognitive parameters in patients after cardiac valve procedures [12–15]. For elderly patients, there is also the option of being allocated to geriatric rehabilitation (GR) instead of indication-specific rehabilitation, if there are at least two different indications to be treated. Therefore, GR mostly accommodates multimorbid patients and aims at the recovery of physical abilities for a largely independent life in the community. The primary aim of GR is to recover an age-appropriate mobility as well as to support self-sufficiency and thus, to avoid long-term care [16–18].

Regarding multimorbid and geriatric patients, the term frailty is often brought up in research, but is not settled as a definite assessment yet. The Valve Academic Research Consortium has underlined the relevance of frailty with defining it as multicomponent including the dimensions of loss of independence, exhaustion, slowness, wasting and malnutrition, poor endurance and inactivity as well as weakness [19]. However, frailty hasn’t been considered as an own cardiovascular risk factor and has also not been included into traditional risk scores like EuroSCORE. Furthermore, several different approaches for measuring frailty have been described. Two indices seem to prevail in clinical studies [20, 21]. Nevertheless, the significance of the indices or its single dimensions for the care pathway after TAVI hasn’t been focused enough in research.

The aim of the study was to characterize older patients after TAVI and to identify predictors of allocating to either geriatric or cardiac rehabilitation under consideration of frailty related components. Our research hypothesis is the following: We assume that patients referred to GR differ to those referred to CR in several physical and psychological domains.

## Methods

### Study setting and participants

In this prospective multicenter cohort study, 635 patients assigned for elective TAVI, which was the only inclusion criterion, were screened in two German heart centers between October 2013 and July 2015. Exclusion criteria were patient refusal, lack of capacity to give informed consent due to poor health status, logistical reasons such as shift of intervention or cancellation of the intervention.

### Baseline measures

Before elective TAVI, sociodemographic data (e. g. age and gender), comorbidities (e. g. stroke/transient ischemic attack [TIA], peripheral artery disease [PAD], diabetes mellitus, coronary artery disease, chronic obstructive pulmonary disease and chronic kidney disease), subjective evaluation of physical activity (volume per week), level of care and echocardiographic parameters (e. g. left ventricular ejection fraction [LVEF] and transaortic gradients) as well as the logistic EuroSCORE were documented in the participating heart centers.

Further, for the quantification of the performance status a standardized 6-min walk test (6MWT) according to current guidelines of the American Thoracic Society [22] based on a distance measuring device was performed. In addition, health related quality of life was assessed by using the questionnaire Short Form 12 (SF-12) [23] with its physical and mental component summaries (PCS and MCS). Anxiety and depression were determined using the Hospital Anxiety and Depression Scale (HADS) [24], and frailty according to the index of Stortecky et al. [25] This Frailty-Index included the Mini Mental State Examination (MMSE), the short form of the Mini Nutritional Assessment Short Form (MNA-SF), Activities of Daily Living (ADL), Instrumental Activities of Daily Living (IADL), Timed Up and Go Test (TUG) and a subjective mobility disability (defined as a decreased frequency of walking 200 m and/or of climbing stairs). The index was summarized with the following allocations: 2 points were assigned if MMSE was < 21 points, and 1 point was assigned for each of the following: MMSE ≥21 and < 27 points, MNA < 12 points, ADL ≥ 1 limited activity, IADL ≥1 limited activity, TUG ≥20 s, and a positive subjective mobility disability. Hence, the Frailty-Index ranged from 0 to 7 points and can be categorized at ≥3 points (frail) vs. < 3 points (non-frail).

The primary endpoint was the assignment to either GR or CR in patients after TAVI, decided by the medical staff in the heart centers. The information was derived on subsequent phone calls with the patients and/or their relatives.

### Statistics

Continuous variables are expressed as means ± standard deviation (SD), and categorical variables as absolute values and percentages. Comparisons between groups were performed using the t-test and the chi-square test, respectively. Predictors of pathways (e. g. GR vs. CR) were identified using a multivariable logistic regression model. We started with a full model containing all available covariates and performed a backwards selection to keep only significant effects in the model. Effects with a *p*-value of less than 0.05 (two-sided) were considered significant. Calculations were carried out using SPSS 25.0 (IBM, Chicago, IL, USA) and Stata (StataCorp. 2017. Stata Statistical Software: Release 15. College Station, TX: StataCorp LLC).

## Results

### Baseline data (Total cohort)

**Fig. 1:**
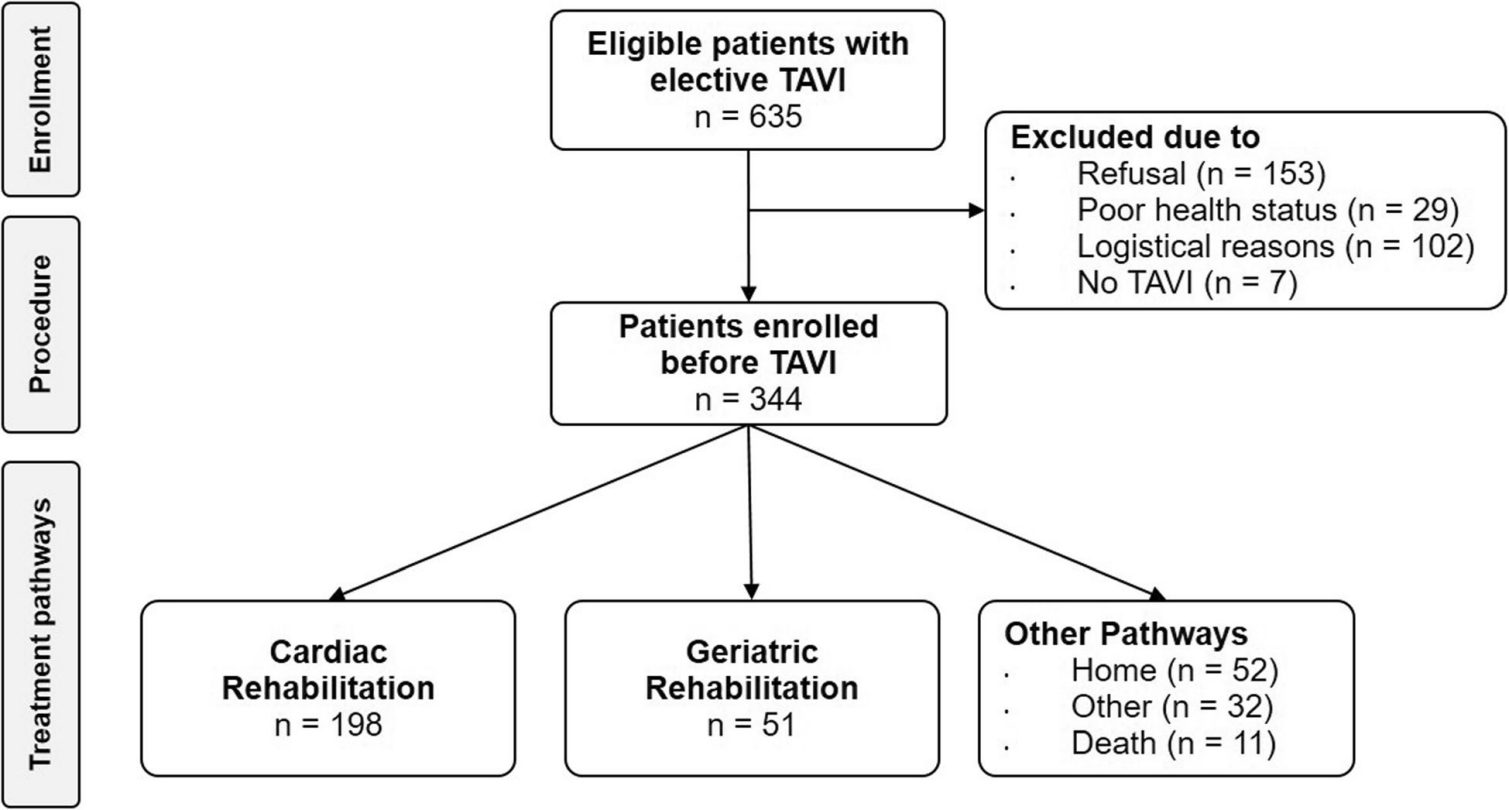
CONSORT flow diagram of inclusion process. TAVI: transcatheter aortic valve implantation

After the exclusion of 291 patients mainly due to patient refusal, 344 patients scheduled for TAVI could be enrolled prior to the procedure. 333 (96.8%) patients were alive at discharge, whereby 198 (59.5%) patients underwent CR after the intervention and 51 (15.3%) patients were allocated to GR. 52 (15.1%) patients rejected rehabilitation and were discharged home (Fig. 1). Thus, data of 249 patients in CR and GR were analyzed.

The patients (mean age 80.7 ± 5.1 years, 147 (59.0%) women) were considered multimorbid with having 2.2 ± 1.3 comorbidities. Moreover, almost half of the patients (42.6%) suffered from diabetes mellitus. Most of the patients (83.1%) did not have a level of care and 110 patients (44.2%) described themselves as very active with more than 150 min of physical activity per week.

Before TAVI, the echocardiographic data showed a mean LVEF of 54.5 ± 10.9% as well as a maximum and mean transvalvular aortic gradient of 71.6 ± 24.9 and 44.9 ± 16.5 mmHg, respectively. Describing the surgical risk, patients showed a logistic EuroSCORE of 16.5 ± 12.1%. (Table 1). Also prior to TAVI, the patients achieved a 6-min walk distance (6MWD) of 239.6 ± 117.9 m and needed a mean walking time of 14.2 ± 7.3 s. in the TUG. With a mean Frailty-Index of 2.4 ± 1.6 points, the investigated population is overall to be classified as non-frail (Table 2).

**Table 1:** Baseline characteristics (Total cohort, cardiac vs. geriatric rehabilitation patients)

	Total cohort (*n* = 249)	CR (*n* = 198)	GR (*n* = 51)	*p*-value
**Patient characteristics**				
Age, years	80.7 ± 5.1	80.3 ± 4.9	82.6 ± 5.4	0.003
Sex, male	102 (41.0)	87 (43.9)	15 (29.4)	0.060
NYHA III/IV	241 (96.8)	190 (96.0)	51 (100.0)	0.145
BMI, kg/m^2^	28.0 ± 4.8	28.0 ± 4.6	27.8 ± 5.4	0.818
Physical activity (subjective)				< 0.001
Inactive (< 90 min/week)	75 (30.1)	45 (22.7)	30 (58.8)	
Active (≥ 90–150 min/week)	64 (25.7)	59 (29.8)	5 (9.8)	
Very active (> 150 min/week)	110 (44.2)	94 (47.5)	16 (31.4)	
Level of care				< 0.001
None	207 (83.1)	181 (91.4)	26 (51.0)	
1	36 (14.5)	16 (8.1)	20 (39.2)	
2	6 (2.4)	1 (0.5)	5 (9.8)	
Diabetes mellitus	106 (42.6)	82 (41.4)	24 (47.1)	0.467
Log. EuroSCORE, %	16.5 ± 12.1	15.0 ± 10.9	22.5 ± 14.6	0.001
Comorbidities, no.	2.2 ± 1.3	2.1 ± 1.2	2.5 ± 1.4	0.051
CAD	159 (63.9)	124 (62.6)	35 (68.6)	0.426
COPD	47 (18.9)	33 (16.7)	14 (27.5)	0.079
PAD	57 (22.9)	37 (18.7)	20 (39.2)	0.002
CKD	114 (45.8)	85 (42.9)	29 (56.9)	0.075
Stroke/TIA	34 (13.7)	30 (15.2)	4 (7.8)	0.175
Length of hospital stay, days	11.1 ± 4.3	10.2 ± 3.8	14.6 ± 4.6	< 0.001
**ECG and Echocardiography**				
Rhythm				0.280
Sinus rhythm	144 (57.8)	119 (60.0)	25 (49.0)	
Atrial fibrillation	98 (39.4)	73 (36.9)	25 (49.0)	
Pacemaker	7 (2.8)	6 (3.1)	1 (2.0)	
LVEF, %	54.5 ± 10.9	55.4 ± 10.3	51.2 ± 12.3	0.027
Left atrium, mm	45.6 ± 6.4	45.4 ± 6.2	46.2 ± 6.8	0.463
LVEDD	48.2 ± 8.3	47.8 ± 8.0	49.6 ± 9.2	0.177
LVPW	13.4 ± 2.9	13.2 ± 2.6	13.7 ± 3.8	0.369
IVS	13.4 ± 2.7	13.4 ± 2.6	13.4 ± 3.1	0.944
Transaortic ∆ P_mean_ (mmHg)	44.9 ± 16.5	44.5 ± 15.8	46.3 ± 19.2	0.544
Transaortic ∆ P_max_ (mmHg)	71.6 ± 24.9	71.1 ± 24.0	73.6 ± 28.2	0.575

Categorical variables are presented in n (%), metric variables in mean ± SD

Abbreviations: *CR*  cardiac rehabilitation, *GR*  geriatric rehabilitation, *COPD*  chronic obstructive pulmonary disease, *PAD*  peripheral artery disease, *CKD*  chronic kidney disease, *TIA*  transient ischemic attack, *ECG*  electrocardiography, *LVEF*  left ventricular ejection fraction, *LVEDD*  left ventricular enddiastolic diameter, *LVPW*  left ventricular posterior wall, *IVS* interventricular septum

**Table 2:** Baseline assessments (Total cohort, cardiac vs. geriatric rehabilitation patients)

Assessments	Total cohort (*n* = 249)	CR (*n* = 198)	GR (*n* = 51)	*p*-value
6MWD, m	239.6 ± 117.9	250.6 ± 115.8	180.3 ± 113.5	0.006
Health related quality of Life				
SF-12 PCS, points	33.1 ± 10.1	33.7 ± 9.8	30.9 ± 11.0	0.085
SF-12 MCS, points	50.5 ± 10.4	50.8 ± 10.5	49.1 ± 9.8	0.285
Emotional Status				
HADS Anxiety, points	5.7 ± 3.9	5.9 ± 3.8	5.9 ± 4.1	0.176
HADS Depression, points	5.6 ± 3.7	5.4 ± 3.5	6.5 ± 4.1	0.050
Frailty-Index, points	2.4 ± 1.6	2.1 ± 1.4	3.8 ± 1.7	< 0.001
Frailty-Index, ≥ 3 pts.	106 (42.6)	67 (33.8)	39 (76.5)	< 0.001
MMSE, points	27.1 ± 2.7	27.3 ± 2.6	26.3 ± 3.3	0.045
MNA-SF, points	11.7 ± 2.3	11.9 ± 2.1	11.1 ± 2.9	0.053
ADL, points	94.2 ± 11.7	97.3 ± 6.8	82.2 ± 17.8	< 0.001
IADL, points	7.0 ± 1.6	7.4 ± 1.1	5.3 ± 2.0	< 0.001
TUG, sec.	14.2 ± 7.3	13.0 ± 6.5	19.0 ± 8.5	< 0.001
Subjective mobility disability	191 (76.7)	150 (75.8)	41 (80.4)	0.485

Categorical variables are presented in n (%), metric variables in mean ± SD

Abbreviations: *CR*  cardiac rehabilitation, *GR*  geriatric rehabilitation, *6MWD*  6-min walk distance, *SF-12*  Short Form 12, *PCS*  physical component summary, *MCS*  mental component summary, *HADS*  Hospital Anxiety and Depression Scale, *MMSE*  Mini Mental State Exam, *MNA-SF*  Mini Nutritional Assessment Short Form, *ADL*  Activities of Daily Living,* IADL*  Instrumental Activities of Daily Living, *TUG*  Timed Up and Go Test

TAVI was performed under a short period of general anesthesia in 83 (33.3%) patients and local anesthesia in 166 (66.7%) patients, whereby the main access route was through the femoral artery in 235 (94.4%) patients and via a left-sided small anterolateral minithoracotomy in 14 (5.6%) patients. A Medtronic CoreValve® Evolut R Prosthesis (Medtronic Inc., Minnesota, USA) was implanted in 165 (66.3%) patients, an Edwards SAPIEN™ transcatheter heart valve (Edwards Lifesciences LLC, Irvine, CA, USA) in 54 (21.7%) patients. After TAVI, the patients stayed in hospital for 11.1 ± 4.3 days.

### Cardiac vs. geriatric rehabilitation patients

In the group comparison, patients allocated to GR were older (82.6 ± 5.4 vs. 80.3 ± 4.9 years; *p* = 0.003) and less physically active (*p* <  0.001) than patients referred to CR. Besides, more of the patients referred to GR had a level of care (*p* <  0.001) and showed a higher surgical risk (22.5 ± 14.6 vs. 15.0 ± 10.9%; *p* = 0.001) before TAVI.

In addition, the patients differed with regard to comorbidities and clinical parameters as more of the GR patients had PAD (39.2 vs. 18.7%; *p* = 0.002) and a significantly lower LVEF (51.2 ± 12.3 vs. 55.4 ± 10.3%; *p* = 0.027). Patients referred to GR stayed in hospital for more days than the CR patients (Table 1).

The groups also varied in functional parameters. The GR patients achieved a lower 6MWD (180.3 ± 113.5 vs. 250.6 ± 115.8 m; *p* = 0.006) as well as a higher depression score (6.5 ± 4.1 vs. 5.4 ± 3.5 points; *p* = 0.050) and a higher overall Frailty-Index (3.8 ± 1.7 [frail] vs. 2.1 ± 1.4 points [non-frail]; *p* <  0.001). Also, the CR and GR patients differed significantly in almost every single component of the Frailty-Index (Table 2).

### Predictors of treatment pathways

In the multivariable regression model, the Frailty-Index as such was not predictive for the treatment pathway. Individual components such as IADL and TUG revealed prognostic impact. Put differently, the chance of being referred to GR was reduced by 51% per one point more in the IADL in favour to CR, while the chance to be referred to GR was 3.97 times higher when the patients needed more or equal to 20 s in the TUG versus the patients who needed less than 10 s.

Similarly, other factors were prognostically relevant. One point more in the PCS of the SF-12 increased the chance for being referred to GR by 7%. The self-assessed physical activity compared to inactivity reduced the probability for GR referral by 79%. Likewise, a patient with level of care 1 had a 4.45 times higher odds for GR than a patient without level of care.

**Fig. 2:**
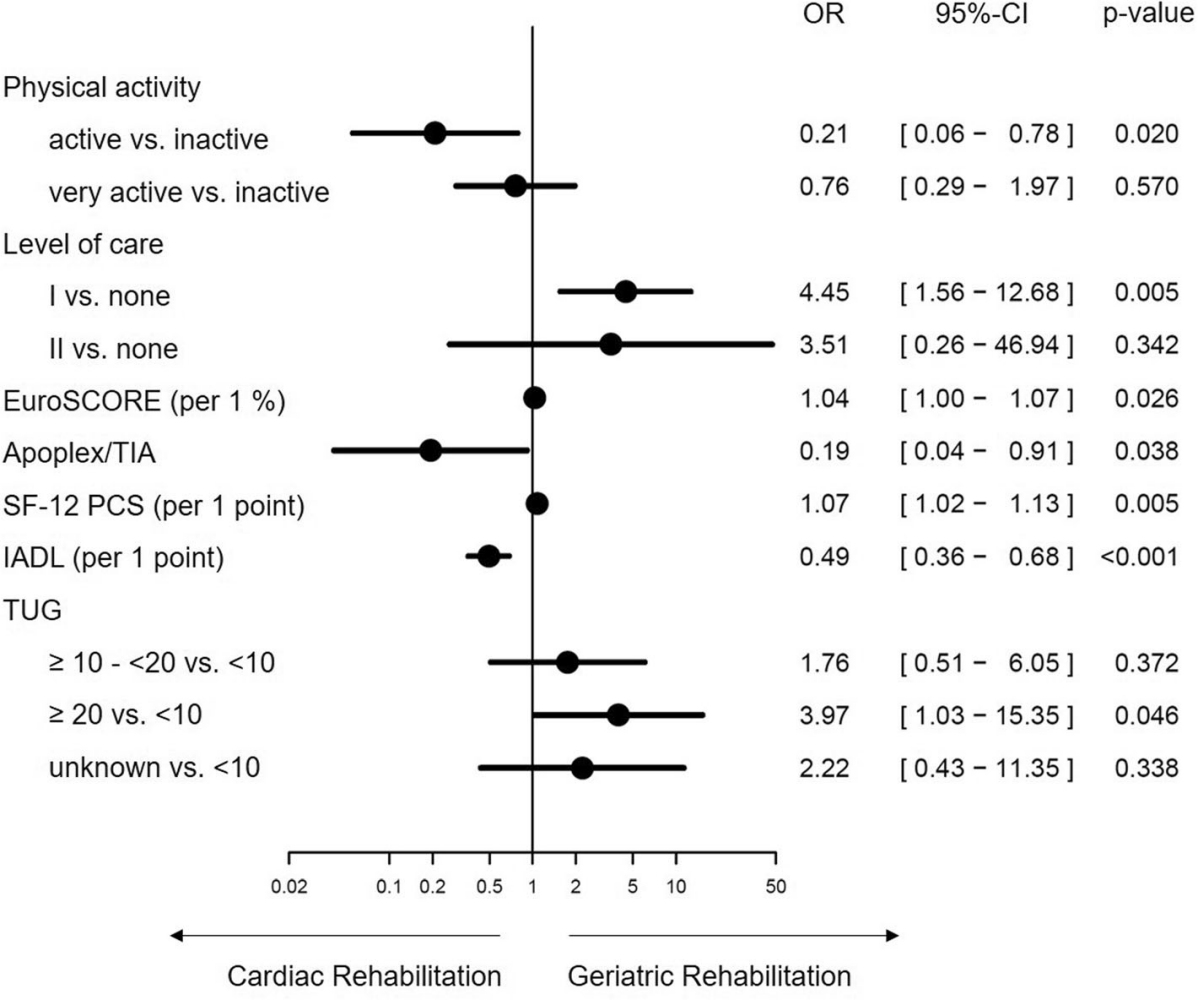
Predictors of treatment pathways in patients after TAVI. OR: Odds Ratio, CI: confidence interval, TIA: transient ischemic attack, SF-12: Short Form 12, PCS: physical component summary, IADL: Instrumental Activities of Daily Living, TUG: Timed Up and Go Test. Apoplex is synonymous with previous stroke. Exemplary explanation: The odds of being referred to geriatric rehabilitation is 4.45 times higher in patients with a level of care 1 versus patients with no level of care.

Finally, clinical parameters were predictive as well. Having had an previous stroke/TIA reduced the chance of being referred to GR by 81%, whereas the odds were 4% higher per 1% more in the logistic EuroSCORE in favour GR (Fig. 2).

## Discussion

In our analysis, we showed that advanced age patients with transcatheter aortic valve implantation differ with regard to on cardiac or geriatric rehabilitation referral. Especially, single components of the Frailty-Index such as lower instrumental activities of daily living and mobility were predictive parameters for the referral to GR. Additionally, health-related quality of life as well as self-assessed physical activity and level of care seem to be of great importance. Also, clinical parameters such as a previous stroke/TIA and the logistic EuroSCORE, initially used to estimate the operative risk in surgical patients, were associated with the referral to cardiac or geriatric rehabilitation.

Concerning the self-assessed level of activity and the level of care, both parameters seem to be conclusive in terms of the decision for CR or GR referral. As the aim of geriatric rehabilitation is the recovery of an age-appropriate mobility and self-sufficiency and thus in the broadest sense the avoidance of long-term care [17], patients who already have a level of care could benefit from this rehabilitation approach.

As for the results of the clinical parameters, the patients who have had a previous stroke were more likely to be assigned to CR, which occurs to be interesting, because we suggested it to be the other way round. It could result from a good recovery and continuous guideline-oriented neurological/cardiological care after the stroke and therefore a good outcome of the intervention. On the contrary, the fact that higher values in the EuroSCORE and therefore a higher surgical risk lead to an enhanced probability of GR referral corresponds to the expectations.

Combining clinical as well as other dimensions, frailty is a geriatric syndrome that is characterized by a vulnerability status with declining function and physiological reserves [26]. Due to the demographic shift, a recent call to action from the European Association of Preventive Cardiology Cardiac Rehabilitation Section promotes the investigation of frail patients also in cardiac rehabilitation settings and recommends to become familiar with some of the tools to recognize and evaluate the severity of this condition [27]. In addition, a recent review concludes that frailty assessments in CR settings should be based on functional, objective tests and should have similar components as tools for risk assessment (e. g. mobility, muscle mass and strength, independence in daily living, cognitive function, nutrition as well as anxiety and depression evaluation) [28].

The results of our study support this importance. The patients were multimorbid with a mean of two comorbidities. There is no doubt that frailty consists of many single domains, but we have to state that the calculation or summary of an overall index for frailty does not seem to be necessary since it doesn’t reveal prognostic impact. Single components such as instrumental activities of daily living or mobility reveal strong prediction on their own for the referral to either geriatric or cardiac rehabilitation and thus for the characterization of this new patient group. This conforms to results of an own study where mobility and nutrition had a prognostic relevance for one-year all-cause mortality in patients after TAVI [29].

Based on our findings, we raise the question if frailty might be more a geriatric than a cardiac rehabilitation topic. Our results show that 34% of the patients undergoing cardiac rehabilitation can be considered frail, whereas 77% of the patients referred to geriatric rehabilitation show a positive frailty according to the index we used. This leads to the assumption that the “real” frail and needy patient does not arrive in CR, but in GR. Consequently, this means that frailty is definitely worth working on in CR, but might be even more important in GR settings. Supporting this thesis, recent research is more and more focusing on investigations of frailty in geriatric rehabilitation and there is an approach to bridge the fields of geriatric medicine and rehabilitation by recognizing the interwoven concepts of multimorbidity, function and frailty [30]. It is also stated that frailty even measured by routinely collected data is feasible and predictive of poor outcomes [31]. Further, other studies about frailty related factors in geriatric rehabilitation conclude that variables such as physical factors such as slow gait speed [32] as well as cognitive and muscular function [33] could be relevant for functional improvement. There is also evidence that geriatric rehabilitation in older patients with cardiovascular disease is feasible [34], whereby the collaboration of geriatric and cardiac rehabilitation scientists and doctors will be a challenging task within the next years. Besides, it is necessary to investigate and to identify the patients that are not frail yet, but might be in a pre-frail state. The upcoming question might be if they are in better hands in either geriatric or cardiac rehabilitation.

Our investigated GR and CR patients also differ in functional parameters such as 6-min walk distance and Timed Up and Go Test. The latter even revealed significance in the multivariable analysis. From the clinical point of view, this could be due to the fact that many GR patients (40% vs. 20% of CR patients) suffered from peripheral artery disease, which could have influenced walking or the movements of the patients. Thus, for achieving results more considerable, other assessments that can be performed independently from the legs must be investigated. Hand grip strength, for instance, can be measured quantitatively using a hand dynamometer. It has already been recommended from the European Working Party on Sarcopenia in Older People and is proposed as a useful assessment of physical performance that is able to determine clinically significant changes [35]. Further, hand grip strength is associated with cardiovascular mortality and can provide valuable prognostic information above and beyond traditional assessments and should therefore be considered for implementation in clinical practice [36].

All in all, the differences between patients referred to cardiac and geriatric rehabilitation setting, respectively, prove the heterogeneity of this population and, finally, the eligibility of both settings. As the new elderly patient group is more diverse than one might think, there should be an overlap between CR and GR. GR should also offer cardiological care and vice versa.

## Limitations

The present study has certain limitations. First, participation was voluntary and thus not without a selection bias, particularly in patients with higher risk profiles, who could have changed the result due to their worse condition after the intervention. It can also be assumed that additionally to this further mentioned selection bias, patients who want to undergo rehabilitation after their clinical stay are different to those who want to go home, who we didn’t consider in our study. Additionally, we do not take into account the post-procedural echocardiographic data regarding the quality of the valve implantation or the different types of valves, which can affect the clinical outcome. Although the underlying Frailty Index captures components such as nutrition, it would be of interest to differentiate between lean and fat body mass as an index of sarcopenia. There are also many indices that consider e.g. serum albumin to have a more differentiated description of the patients. This would require a further approach to characterizing TAVI patients and may be advocated for detailed research.

## Conclusion

Advanced age patients after transcatheter aortic valve implantation referred to cardiac or geriatric rehabilitation differ in single components of the Frailty-Index such as instrumental activities of daily living and mobility were objected as predictive parameters for the subsequent post-interventional care. Additionally, the evaluation of health related quality of life as well as self-evaluated physical activity and level of care seem to be of great importance. Also, clinical parameters such as a previous stroke/TIA and the logistic EuroSCORE were associated with the assignment. The patients seem to be different with specific needs. Thus, our data prove the eligibility of both CR and GR settings.

## Data Availability

The datasets used and/or analyzed during the current study are available from the corresponding author on reasonable request. All data generated or analyzed during this study are included in this published article.

## Abbreviations

AS: Aortic stenosis
6MWD: 6-min walk distance
6MWT: 6-min walk test
ADL: Activities of daily living,
CR: Cardiac rehabilitation
GR: Geriatric rehabilitation
HADS: Hospital anxiety and depression scale
IADL: Instrumental activities of daily living
LVEF: Left ventricular ejection fraction
MCS: Mental component summary
MMSE: Mini mental state examination
MNA-SF: Mini nutritional assessment short form
OR: Odds ratio
PAD: Peripheral artery disease
PCS: Physical component summary
SD: Standard deviation
SF-12: Short form 12
TAVI: Transcatheter aortic valve implantation
TIA: Transient ischemic attack
TUG: Timed up and go test
